# The Downregulation of eIF3a Contributes to Vemurafenib Resistance in Melanoma by Activating ERK via PPP2R1B

**DOI:** 10.3389/fphar.2021.720619

**Published:** 2021-08-27

**Authors:** Shi-Long Jiang, Zhi-Bin Wang, Tao Zhu, Ting Jiang, Jiang-Feng Fei, Chong Liu, Chao Luo, Yan Cheng, Zhao-Qian Liu

**Affiliations:** ^1^ Department of Clinical Pharmacology, Hunan Key Laboratory of Pharmacogenetics, and National Clinical Research Center for Geriatric Disorders, Xiangya Hospital, Central South University, Changsha, China; ^2^ Institute of Clinical Pharmacology, Engineering Research Center for Applied Technology of Pharmacogenomics of Ministry of Education, Central South University, Changsha, China; ^3^ Department of Pharmacy, The Second Xiangya Hospital, Central South University, Changsha, China; ^4^ Sinocare Inc., Changsha, China; ^5^ Shanghai Mental Health Center, Shanghai JiaoTong University School of Medicine, Shanghai, China

**Keywords:** melanoma, vemurafenib resistance, eIF3a, ERK, PPP2R1B

## Abstract

Vemurafenib, a BRAF V600E inhibitor, provides therapeutic benefits for patients with melanoma, but the frequent emergence of drug resistance remains a challenge. An understanding of the mechanisms underlying vemurafenib resistance may generate novel therapeutic strategies for patients with melanoma. Here, we showed that eIF3a, a translational regulatory protein, was an important mediator involved in vemurafenib resistance. eIF3a was expressed at significantly lower levels in vemurafenib-resistant A375 melanoma cells (A375R) than in parental A375 cells. Overexpression of eIF3a enhanced the sensitivity to BRAF inhibitors by reducing p-ERK levels*.* Furthermore, eIF3a controlled ERK activity by regulating the expression of the phosphatase PPP2R1B via a translation mechanism, thus determining the sensitivity of melanoma cells to vemurafenib. In addition, a positive correlation between eIF3a and PPP2R1B expression was also observed in tumor samples from the Human Protein Atlas and TCGA databases. In conclusion, our studies reveal a previously unknown molecular mechanism of BRAF inhibitor resistance, which may provide a new strategy for predicting vemurafenib responses in clinical treatment.

## Introduction

Melanoma is an aggressive cancer with a rapidly increasing incidence (Bellmann et al., 2020). Approximately 60% of melanoma patients harbored BRAF kinase mutation (BRAFV600E), which activates the MAPK pathway and contributes to the immortalization of cancer (Betancourt et al., 2019). Selective BRAF inhibitors (BRAFis), such as vemurafenib, have been approved for clinical use and significantly improved the progression-free survival (PFS) and overall survival (OS) of patients with melanoma carrying BRAF mutations compared with the traditional chemotherapy dacarbazine (Chapman et al., 2011; Kim and Cohen, 2016; Chapman et al., 2017). However, the universal emergence of resistance after vemurafenib treatment limits its application in the clinic (Sosman et al., 2012; Sullivan and Flaherty, 2013; Samatar and Poulikakos, 2014; Garbe and Eigentler, 2018). Multiple mechanisms involved in vemurafenib resistance have been identified (Sullivan and Flaherty, 2013); however, these mechanisms do not fully characterize the causes of vemurafenib resistance, and effective targets and strategies for overcoming clinical vemurafenib resistance are lacking, encouraging further research (Rizos et al., 2014).

Eukaryotic translation initiation factor 3a (eIF3a), the largest subunit of the eIF3 complex, plays an important role in interacting with and recruiting mRNA to the ribosome (Zhang et al., 2007; Saletta et al., 2010; Wang et al., 2016). eIF3a expression is associated with physiological and pathological processes by regulating the cell cycle, apoptosis, differentiation and fibrosis (Dong et al., 2009; Zhang Y. F. et al., 2015; Wang et al., 2016; Yin et al., 2018). Many researchers have reported an important role for eIF3a in the occurrence and development of tumors, and elevated eIF3a expression favors the maintenance of the tumor malignant phenotype (Yin et al., 2011a; Jiang et al., 2020). Recent reports suggested that eIF3a could mediate glycolytic metabolism, and the level of anti-eIF3A autoantibody in serum may represent a potential diagnostic marker for hepatocellular carcinoma (Heo et al., 2019; Miao et al., 2019). Moreover, many studies have suggested that eIF3a affects patient prognosis and treatment responses; for example, patients with cancer presenting high eIF3a levels experience better relapse-free and overall survival than those with low eIF3a expression (Dellas et al., 1998; Chen and Burger, 1999). Previous studies, including our own study, supported the hypothesis that eIF3a knockdown reduces the cellular response to cisplatin by regulating the expression of DNA repair proteins in lung cancer (Yin et al., 2011a), nasopharyngeal carcinoma (Liu et al., 2011), and ovarian cancer (Zhang Y. et al., 2015). Tumia R et al. also reported that high eIF3a expression increases radiotherapy and chemotherapy responses in patients with breast, gastric, lung, and ovarian cancer (Tumia et al., 2020), further suggesting that eIF3a may affect patient responses to treatments.

Here, this study reported for the first time that eIF3a expression is positively correlated with vemurafenib sensitivity in melanoma cells, and this effect of eIF3a is mediated by regulating the translation of the protein phosphatase PPP2R1B, which inhibits ERK phosphorylation. Furthermore, the relationship between eIF3a and PPP2R1B expression was detected in samples from patients with cancer. These findings not only reveal a novel role for eIF3a in vemurafenib resistance and present eIF3a/PPP2R1B/ERK as a new regulatory pathway in cancer but also may provide a novel biomarker for predicting the vemurafenib response.

## Materials and Methods

### Cell Lines and Culture

The human malignant melanoma cell lines A375 and SK-28 were purchased from ATCC (Manassas, VA, United States). Vemurafenib-resistant A375 cells (A375R) were established in our lab. HEK293T cells were maintained in our lab. A375, SK-28, and HEK293T cells were cultured in high-glucose DMEM. A375R cells were cultured in DMEM supplemented with 2 μM vemurafenib. All cell culture media were supplemented with 10% fetal bovine serum, 100 units/ml penicillin and 100 μg/ml streptomycin. All cell lines were maintained at 37°C in a humidified atmosphere containing 5% CO_2_/95% air. All cells were confirmed negative for *mycoplasma* using a *mycoplasma* detection kit.

### Reagents and Antibodies

Vemurafenib, dabrafenib and L-mimosine were purchased from Selleck (Shanghai, China). The ERK inhibitor SCH772984 was purchased from TOPSCIENCE (Shanghai, China). The anti-eIF3a antibody (cat. no. ab128996, 1:1,000) and anti-PPP2R1B antibody (cat. no. ab154815, 1:1,000) were purchased from Abcam. The anti-ERK antibody (cat. no. 4695, 1:1,000) and anti-p-ERK antibody (Thr202/Tyr204) (cat. no. 4370, 1:1,000) were purchased from Cell Signaling Technologies. The anti-puromycin antibody (cat. no. EQ0001, 1:1,000) was purchased from Kerafast. The anti-Flag antibody (cat. no. M185, 1:1,000) and anti-HA antibody (cat. no. M180, 1:1,000) were purchased from MBL. β-action (1:10,000) and β-Tubulin (1:10,000) antibodies were purchased from Proteintech. An enhanced chemiluminescence (ECL) kit was purchased from Cytiva. CCK8 was purchased from Bimake (Shanghai, China).

### siRNA and Plasmid Transfection

The siRNAs targeting eIF3a and PPP2R1B were purchased from RiboBio (Guangzhou, China). The PPP2R1B plasmid was purchased from Gene (Shanghai, China). Transfection of siRNAs was performed according to the manufacturer’s protocol. Briefly, cells in the exponential phase of growth were plated in six-well tissue culture plates and then transfected with siRNAs using Lipofectamine RNAimax (Invitrogen) reagent and OPTI-MEM medium. The plasmid was transfected using the Lipofectamine 2000 (Invitrogen) reagent according to the manufacturer’s protocol.

### Western Blotting Analysis

After treatment, the cells were lysed on ice for 30 min in RIPA buffer supplemented with protease inhibitor and phosphatase inhibitor cocktails A and B (Biotool), followed by centrifugation at 12,000 × g for 15 min at 4°C. The protein concentration of the supernatant was determined using a BCA assay (Beyotime Biotechnology, Shanghai, China). Proteins were resolved on SDS-PAGE gels and then transferred to PVDF membranes (0.22 µm, Merck Millipore). After blocking with skim milk, the PVDF membranes were incubated with the respective antibodies in 5% BSA at 4°C overnight, followed by an incubation with a secondary antibody at room temperature for 1 h. The protein signals were detected using an enhanced chemiluminescence kit.

### Clonogenic Assay

Cells were plated in 6-well tissue culture plates (1,000 cells per well) and exposed to the indicated treatment at 37°C in a humidified atmosphere containing 5% CO_2_/95% air. At the end of the incubation, cells were fixed with 4% paraformaldehyde, stained with crystal violet for 30 min, and washed with PBS, and then the colonies were photographed and counted.

### Acquisition and Analysis of GEO Data

Gene expression data from GSE118239 were extracted from the NCBI Gene Expression Omnibus (GEO) database. The Gene Set Enrichment Analysis (GSEA) was completed using the R package, “clusterProfiler”.

### Cell Viability Assay

Cell viability was measured using CCK8 (Bimake, Shanghai, China) reagent. Briefly, 2 × 10^3^ cells/well were plated in each well of 96-well plates and treated with various drug concentrations for the indicated durations. After treatment, 10 μl of CCK8 reagent were added to each well and incubated for 1 h. The absorbance was read at a 450 nm wavelength.

### Quantitative Real-Time PCR

Total RNA was isolated from cells using TRIzol reagent (Takara), and 1 μg of total RNAs was reverse transcribed using the PrimeScript RT Reagent Kit (Perfect real time) (Takara). Real-time PCR was performed using SYBR Premix Ex Tap (Takara) and run on an LC480 instrument. For the quantification of gene expression, the 2^−ΔΔCt^ method was used. The actin expression level was used for normalization.

### Immunofluorescence Staining

Cells were transfected with the eIF3a siRNA or a control siRNA. After transfection, the cells were washed twice with ice-cold PBS, fixed with 4% paraformaldehyde for 30 min and treated with 0.5% Triton-100X for 10 min. The cells were blocked at room temperature for 60 min and incubated with a p-ERK antibody overnight at 4°C. Cells were then incubated with a secondary antibody at room temperature for 1 h. The cell nuclei were stained with DAPI for 2 min. Finally, p-ERK levels were observed with a fluorescence microscope.

### Luciferase Reporter Assay

To analyze PPP2R1B translation activity, eIF3a-silenced 293T cells were cotransfected with the PPP2R1B-containing luciferase reporter plasmid. Cells were harvested, and the luciferase activities were measured using the Promega Luciferase Assay System (Promega, Madison, Wisconsin).

### RNA Immunoprecipitation

The RIP assay was performed according to the manufacturer’s instructions using an EZMagna RIP Kit (Millipore, Billerica, MA). Briefly, 293T cells lysates were incubated with anti-eIF3a antibodies or anti-IgG at 4°C overnight. Next, the expression of the PPP2R1B mRNA was determined using qRT-PCR.

### Metabolic Labeling With Puromycin

Protein synthesis activity was assessed using puromycin labeling. Briefly, cells were transfected with the eIF3a siRNA or non-targeting RNA. After transfection, the cells were incubated with puromycin (10 mg/ml) for 30 min. Cells were lysed at 4°C, and the protein concentration was determined using a BCA assay. Equal amounts of protein were resolved on SDS-PAGE gels and then transferred to PVDF membranes. Signals were detected with an anti-puromycin antibody.

### Statistical Analysis

All experiments were performed at least three times. For measurements of CCK8 assays, statistical analyses were performed using Student’s *t-test* with GraphPad Prism software. *p* values <0.05 were considered statistically significant.

## Results

### eIF3a is Associated With Vemurafenib Sensitivity

**FIGURE 1:**
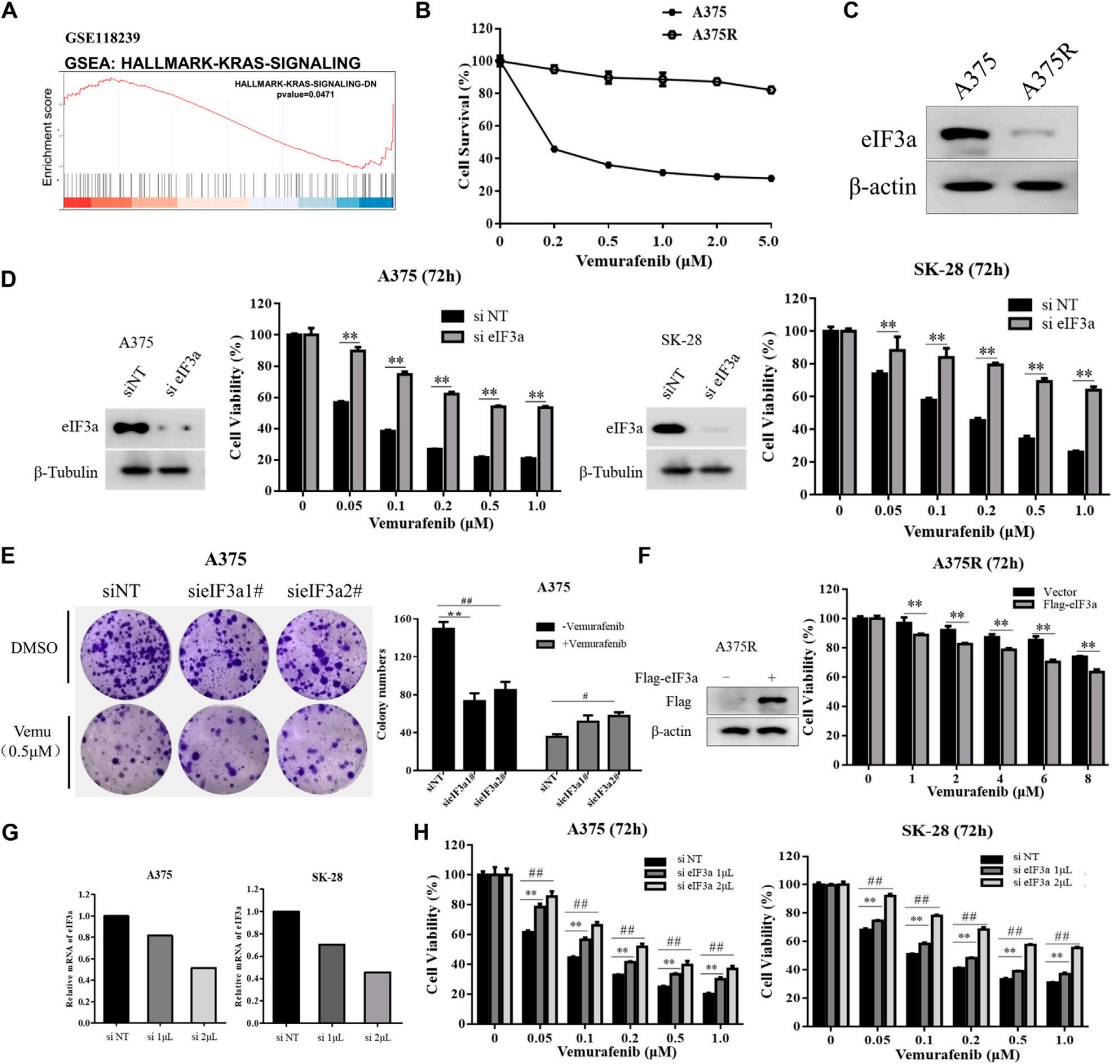
eIF3a is associated with vemurafenib sensitivity. **(A)** Enrichment plots from the gene set enrichment analysis (GSEA) of eIF3a in the KRAS signaling pathway. **(B)** A375 and A375R cells were treated with a series of vemurafenib concentrations for 72 h, cell survival was measured using the CCK8 assay. **(C)** The expression of eIF3a in A375 and A375R cells was measured using western blotting. **(D)** A375 and SK-28 cells were transfected with the non-targeting RNA or eIF3a siRNA and then treated with vemurafenib for 72 h, cell viability was measured by the CCK8 assay. ^**^ *p* < 0.01, *t-test*. **(E)** A375 cells were transfected with eIF3a siRNA and treated with vemurafenib, cell proliferation was measured by the colony formation assay. ^*/#^ *p* < 0.05, ^**/##^ *p* < 0.01, *t-test*. **(F)** A375R cells transfected with the empty vector or Flag-eIF3a plasmid and then treated with vemurafenib for 72 h, cell viability was measured by the CCK8 assay. ^**^ *p* < 0.01, *t-test*. **(G,H)** A375 and SK-28 cells were transfected with a non-targeting siRNA or an eIF3a siRNA with the indicated concentrations, then treated with vemurafenib for 72 h, cell viability was measured by the CCK8 assay. ^**/##^ *p* < 0.01, *t-test*.

To explore the potential function of eIF3a in cancer therapy, gene set enrichment analysis (GSEA) was used for pathway enrichment analysis. The KEGG analysis showed that the KRAS signaling pathway was significantly enriched after knockdown of eIF3a (GSE118239) (Figure 1A). Reactivation of RAS is associated with vemurafenib resistance (Boussemart et al., 2014), therefore, we hypothesize that eIF3a may be involved in vemurafenib resistance. To test this hypothesis, we first compared the levels of eIF3a in A375 and A375R cells, which is less responsive to vemurafenib than A375 cells (Figure 1B) and observed a significant downregulation of eIF3a in A375R cells (Figure 1C). Furthermore, siRNA-mediated knockdown of eIF3a decreased the responses of human melanoma cells to vemurafenib, as evidenced by the results of the CCK8 assay and colony formation assay (Figures 1D,E). Furthermore, overexpression of eIF3a rendered A375R cells more sensitive to vemurafenib (Figure 1F). To further support the idea that eIF3a is associated with vemurafenib sensitivity, A375 and SK-28 cells were transfected with different amounts of siRNA, followed by vemurafenib treatment. Figures 1G,H showed that the level of eIF3a was positively correlated with vemurafenib sensitivity. These data suggest that the reduced expression of eIF3a in melanoma may result in vemurafenib resistance.

**FIGURE 2:**
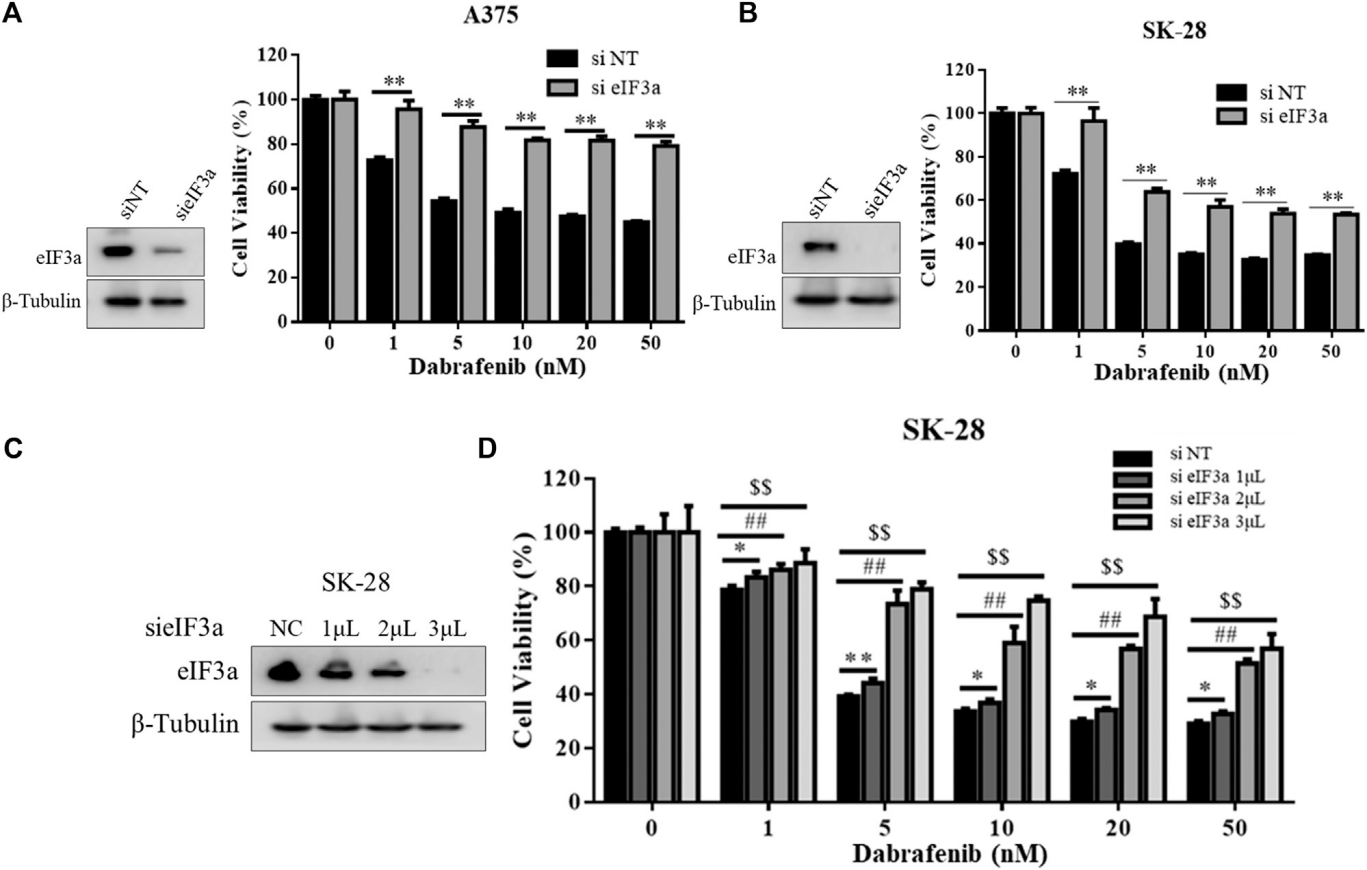
eIF3a was also associated with dabrafenib sensitivity. **(A,B)** A375 and SK-28 cells were transfected with the non-targeting RNA or eIF3a siRNA and then treated with dabrafenib for 72 h, cell viability was measured by the CCK8 assay. ^**^ *p* < 0.01, *t-test*. **(C)** SK-28 cells were transfected with a non-targeting siRNA or an eIF3a siRNA with the indicated concentrations, the expression of eIF3a was measured by western blotting. **(D)** SK-28 cells were transfected with the indicated concentrations of a non-targeting siRNA or an eIF3a siRNA, and then treated with dabrafenib for 72 h, cell viability was measured using the CCK8 assay. ^*/#/$^ *p* < 0.05, ^**/##/$$^ *p* < 0.01, *t-test*.

In addition, we also evaluated the effect of eIF3a on the antitumor activity of dabrafenib, another BRAF inhibitor approved by the FDA to treat melanoma (Puszkiel et al., 2019). Consistent with the results obtained with vemurafenib, eIF3a silencing also reduced the cytotoxicity of dabrafenib (Figures 2A,B), and eIF3a expression was positively correlated with the toxicity of dabrafenib (Figures 2C,D). These experiments confirmed the involvement of eIF3a in regulating the sensitivity of human melanoma cells to dabrafenib.

### eIF3a Alters the Sensitivity of Melanoma Cells to Vemurafenib by Controlling ERK Signaling

**FIGURE 3:**
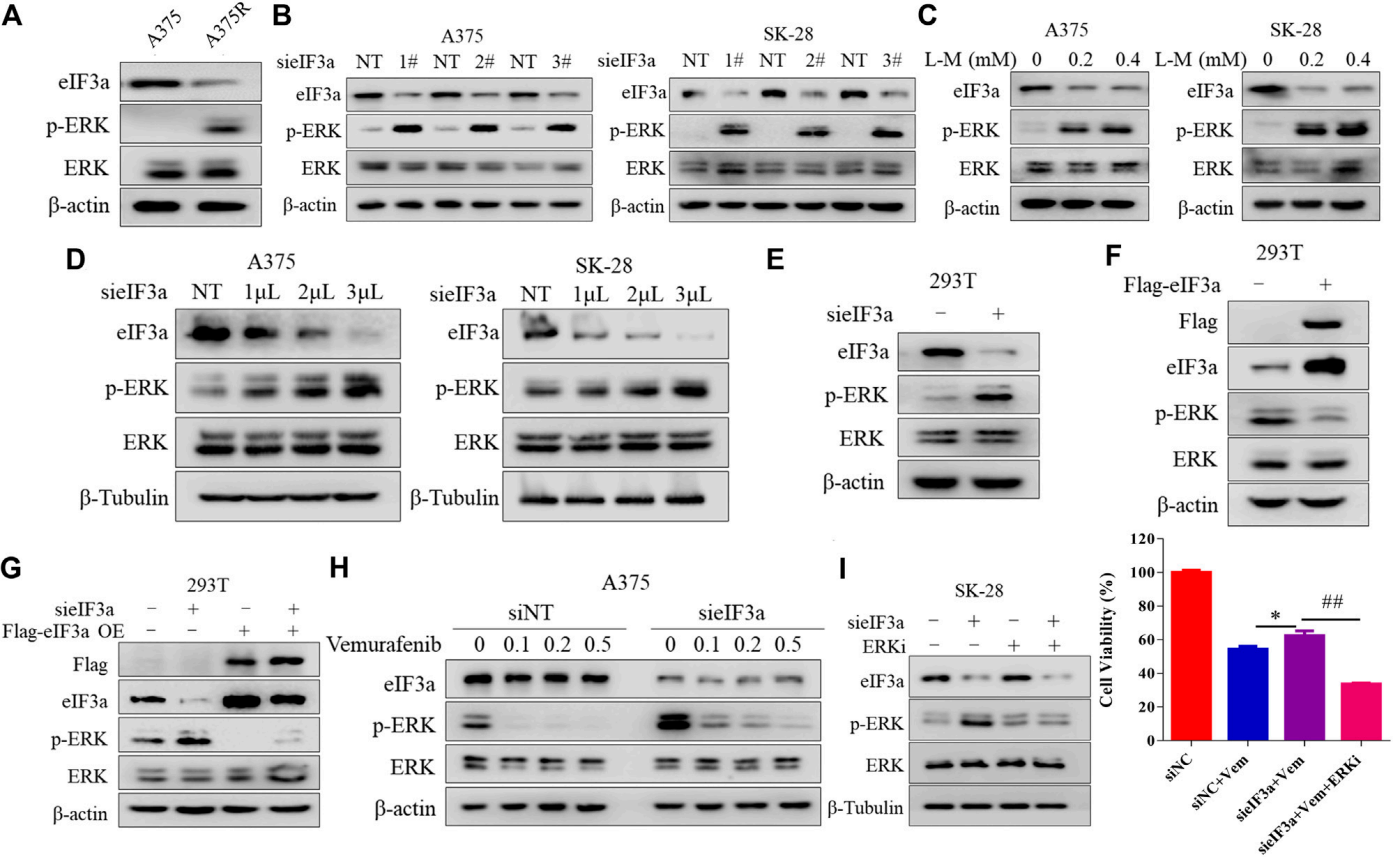
eIF3a alters the sensitivity of melanoma cells to vemurafenib by controlling ERK signaling. **(A)** The levels of eIF3a, p-ERK and ERK in A375 and A375R cells were measured using western blotting. **(B)** A375 and SK-28 cells were transfected with a non-targeting siRNA or an eIF3a siRNA, the levels of eIF3a, p-ERK and ERK were measured using western blotting. **(C)** A375 or SK-28 cells were treated with the indicated concentrations of the eIF3a inhibitor L-mimosine, and the levels of eIF3a, p-ERK and ERK were measured using western blotting. **(D)** A375 and SK-28 cells were transfected with the indicated concentrations of a non-targeting siRNA or an eIF3a siRNA, and the levels of eIF3a, p-ERK and ERK were measured using western blotting. **(E)** HEK293T cells were transfected with a non-targeting siRNA or an eIF3a siRNA, and the levels of eIF3a, p-ERK and ERK were measured using western blotting. **(F)** HEK293T cells were transfected with a control plasmid or a Flag-eIF3a plasmid, and the levels of eIF3a, p-ERK and ERK were measured using western blotting. **(G)** HEK293T cells were transfected with a non-targeting siRNA or an eIF3a siRNA, followed by transfection with the Flag-eIF3a plasmid. The levels of Flag, eIF3a, p-ERK and ERK were measured using western blotting. **(H)** A375 cells were transfected with the non-targeting RNA or eIF3a siRNA and then treated with vemurafenib. The levels of eIF3a, p-ERK and ERK were measured using western blotting. **(I)** SK-28 cells were transfected with a non-targeting siRNA or an eIF3a siRNA, and then treated with vemurafenib alone or in combination with an ERK inhibitor. Cell viability was measured using the CCK8 assay. ^*^ *p* < 0.05, ^##^ *p* < 0.01, *t-test*.

Next, we investigated the molecular mechanism by which eIF3a regulates sensitivity to vemurafenib. Figure 1A shows that inhibiting eIF3a activated the RAS signaling pathway. It’s well known that ERK is the classical downstream target of the RAS signaling pathway, and the aberrant activation of ERK plays a central role in vemurafenib resistance (Davies et al., 2002). Consistent with this finding, we also verified that the A375R cells harbored higher p-ERK and lower eIF3a levels than the A375 cells (Figure 3A). Therefore, we next determined whether eIF3a regulates the sensitivity of melanoma cells to vemurafenib by modulating ERK activity. As expected, inhibiting eIF3a with either siRNA or L-mimosine, a small-molecule inhibitor of eIF3a, significantly increased ERK phosphorylation in melanoma cells (Figures 3B,C). Figures 3D,E further shows the negative correlation between the expression of eIF3a and p-ERK. Immunofluorescence staining was also used to detect p-ERK levels after silencing eIF3a (Supplementary Figures S1A,B). Moreover, the regulation of p-ERK by eIF3a was also observed in non-small cell lung cancer and breast cancer cell lines (Supplementary Figures S1C,D). Ectopic expression of eIF3a resulted in a reduction in ERK phosphorylation (Figure 3F). We reintroduced eIF3a into eIF3a knockdown cells by transfecting an eIF3a overexpression plasmid and then measured p-ERK levels to further validate the effect of eIF3a on ERK activation. As shown in Figure 3G, p-ERK levels were increased in the cells with eIF3a knockdown, and eIF3a overexpression blocked the silencing expression. Furthermore, knockdown of eIF3a in A375 cells mitigated the suppressive effects of vemurafenib on p-ERK levels, revealing that the vemurafenib-induced MAP kinase signaling blockade was attenuated by silencing eIF3a (Figure 3H). And by combination with an ERK inhibitor, the insensitivity to vemurafenib after knockdown of eIF3a was overcome (Figure 3I). Based on these results, the loss of eIF3a contributed to ERK activation, in turn conferring BRAF inhibitor resistance.

### eIF3a Suppresses ERK Activity by Upregulating the Expression of PPP2R1B

**FIGURE 4:**
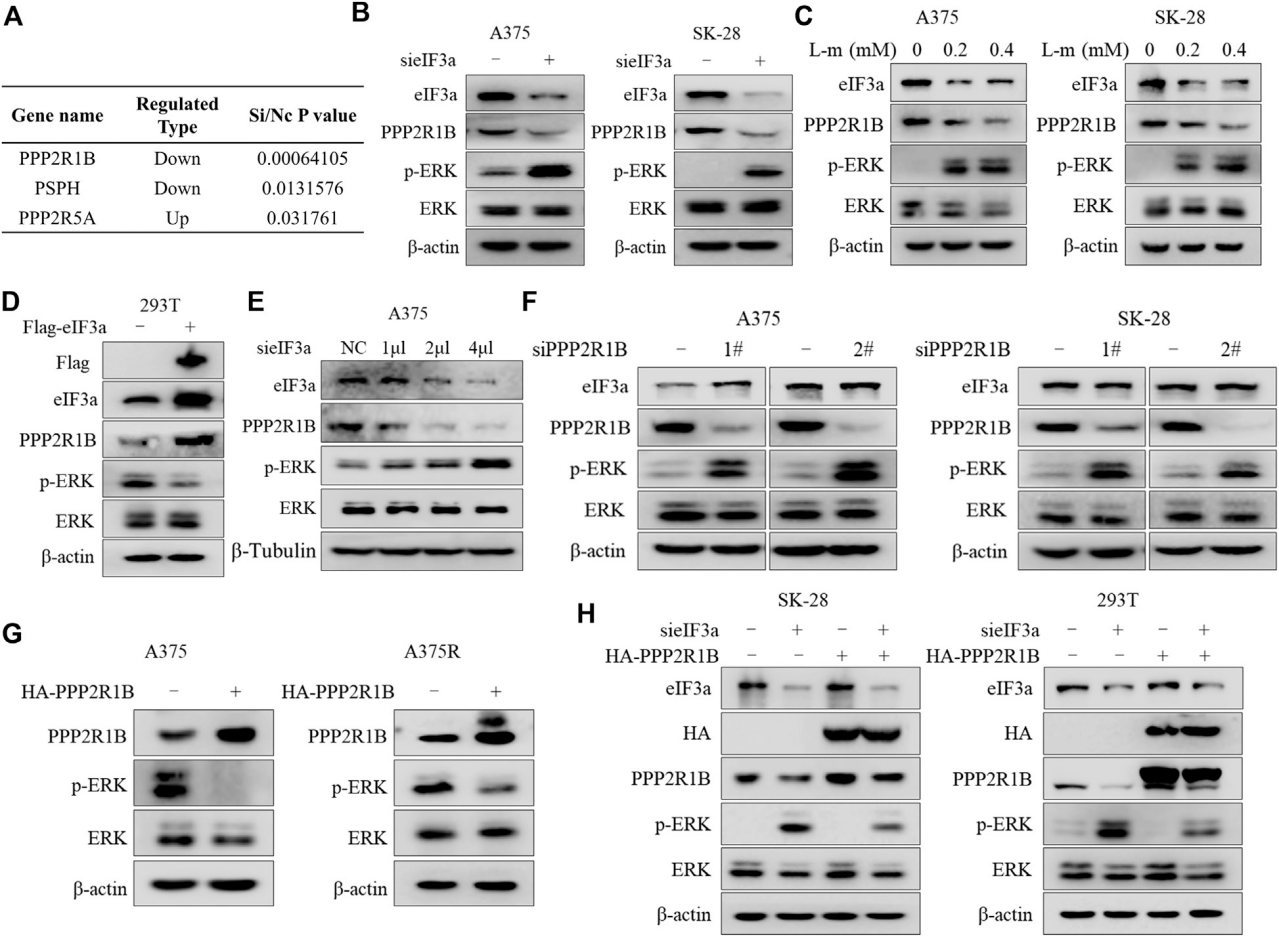
eIF3a suppresses ERK activity by upregulating the expression of PPP2R1B. **(A)** Differential expression of three protein phosphatases was observed after silencing eIF3a using mass spectrometry. **(B)** A375 and SK-28 cells were transfected with a non-targeting siRNA or an eIF3a siRNA, and the levels of eIF3a, PPP2R1B, p-ERK and ERK were measured using western blotting. **(C)** A375 and SK-28 cells were treated with the indicated concentrations of the eIF3a inhibitor L-mimosine, and the levels of eIF3a, PPP2R1B, p-ERK and ERK were measured using western blotting. **(D)** HEK293T cells were transfected with a control plasmid or a Flag-eIF3a plasmid, and the levels of Flag, eIF3a, PPP2R1B, p-ERK and ERK were measured using western blotting. **(E)** A375 cells were transfected with the indicated concentrations of a non-targeting siRNA or an eIF3a siRNA, and the levels of eIF3a, PPP2R1B and p-ERK were measured using western blotting. **(F)** A375 and SK-28 cells were transfected with a non-targeting siRNA or PPP2R1B siRNA, and the levels of eIF3a, PPP2R1B, p-ERK and ERK were measured using western blotting. **(G)** A375 and A375R cells were transfected with a control plasmid or a HA-PPP2R1B plasmid, and the levels of PPP2R1B, p-ERK and ERK were measured using western blotting. **(H)** SK-28 or HEK293T cells were transfected with a non-targeting siRNA or an eIF3a siRNA, followed by transfection with a HA-PPP2R1B plasmid. The levels of eIF3a, HA, PPP2R1B, p-ERK and ERK were measured using western blotting.

We next sought to investigate the molecular mechanism underlying eIF3a knockdown-mediated ERK activation. The MAPK pathway is dephosphorylated by numerous protein phosphatases, for example, reductions in the levels of dual specificity phosphatase 4 (DUSP4) and dual specificity phosphatase 6 (DUSP6) result in activation of the MAP kinase pathway (Kondoh and Nishida, 2007; Kidger and Keyse, 2016; Gupta et al., 2019). Therefore, we wondered whether a phosphatase was involved in regulating eIF3a knockdown-mediated ERK activation. We first measured the expressions of protein phosphatases after silencing eIF3a using mass spectrometry to assess this hypothesis. Among the phosphatases examined, the expression of PPP2R1B, a regulatory subunit of the PP2A complex (Hamano et al., 2011), was decreased in eIF3a-knockdowned cells compared with that in control cells (Figure 4A).

Consistent with the proteomics results, knockdown or inhibition of eIF3a with an siRNA or small-molecule inhibitor decreased PPP2R1B expression and increased p-ERK levels (Figures 4B,C), and overexpression of eIF3a resulted in PPP2R1B elevation (Figure 4D). Figure 4E further demonstrated that eIF3a could regulate PPP2R1B expression. In addition, the regulatory effect of eIF3a on PPP2R1B protein levels was also determined in non-small cell lung cancer cells and breast cancer cells (Supplementary Figure S2). Next, we examined whether a regulatory effect existed between PPP2R1B and ERK. As shown in Figure 4F, PPP2R1B silencing caused a significant increase in p-ERK levels, and overexpression of PPP2R1B induced a remarkable reduction in p-ERK levels (Figure 4G). We overexpressed PPP2R1B after silencing eIF3a to further determine whether PPP2R1B mediates the regulatory effect of eIF3a on ERK activity. Figure 4H shows that reintroduction of PPP2R1B reversed the upregulation of p-ERK induced by eIF3a deficiency.

We further investigated the functional role of PPP2R1B in vemurafenib resistance, and found that PPP2R1B knockdown reduced the sensitivity of A375 cells to vemurafenib, whereas overexpression of PPP2R1B significantly enhanced the antitumor effect of vemurafenib (Supplementary Figures S3A,B). Furthermore, ectopic expression of PPP2R1B in eIF3a-silenced cells partially restored the sensitivity to vemurafenib (Supplementary Figure S3C). These findings support the hypothesis that eIF3a controls the action of ERK by regulating PPP2R1B expression, thus determining the sensitivity of melanoma cells to vemurafenib.

### eIF3a Upregulates the Expression of PPP2R1B by Promoting its Translation

**FIGURE 5:**
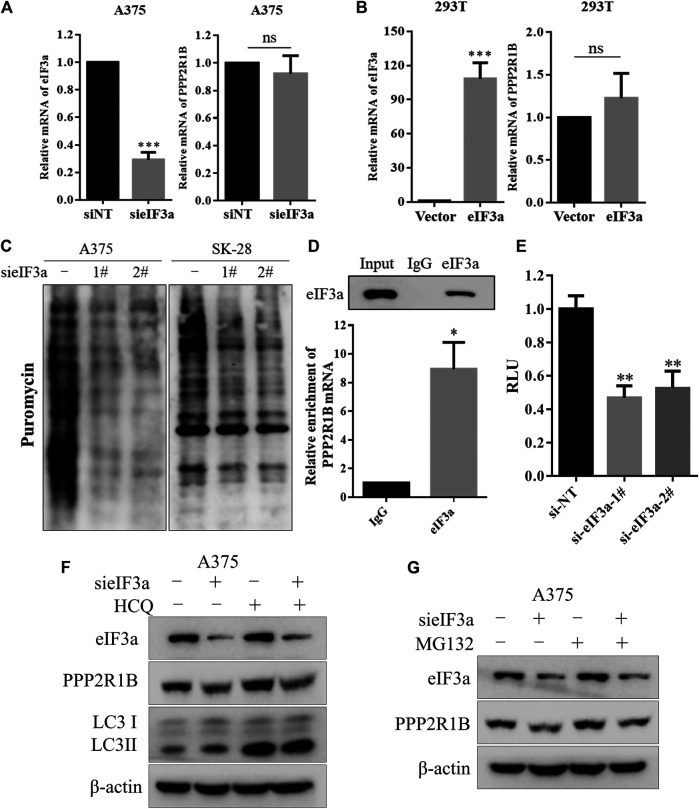
eIF3a upregulates the expression of PPP2R1B by promoting its translation. **(A)** A375 cells were transfected with a non-targeting siRNA or an eIF3a siRNA, and levels of the eIF3a and PPP2R1B mRNAs were measured using qRT-PCR. **(B)** HEK293T cells were transfected with a control plasmid or a Flag-eIF3a plasmid, and the expression of the eIF3a and PPP2R1B mRNAs was measured using qRT-PCR. **(C)** A375 or SK-28 cells were transfected with a non-targeting siRNA or an eIF3a siRNA, followed by treatment with puromycin for 30 min. Puromycin incorporation was measured using western blotting. **(D)** RIP assay indicating that the PPP2R1B mRNA bound to the eIF3a protein. **(E)** Luciferase assay showing significantly lower activity of the PPP2R1B reporter construct after eIF3a knockdown in HEK293T cells. **(F)** A375 cells were transfected with a non-targeting siRNA or an eIF3a siRNA, followed by treatment with 20 μM HCQ for 24 h. The levels of eIF3a, PPP2R1B and LC3 were measured using western blotting. **(G)** A375 cells were transfected with a nontargeting siRNA or an eIF3a siRNA, followed by treatment with 20 μM MG132 for 4 h. The levels of eIF3a and PPP2R1B were measured using western blotting.

Next, we wanted to explore how eIF3a affects PPP2R1B expression. We examined the PPP2R1B mRNA level after knockdown or overexpression of eIF3a. Silencing or overexpression of eIF3a did not change the PPP2R1B mRNA level (Figures 5A,B), indicating that eIF3a regulated PPP2R1B at the posttranscriptional level. Because eIF3a is a translation regulator, we monitored the role of eIF3a in ongoing translational regulation by measuring protein synthesis using puromycin labeling. Figure 5C showed that the translational activity was reduced after eIF3a knockdown, indicating that eIF3a is indeed involved in protein synthesis in cells. In addition, the PPP2R1B mRNA could bind to the eIF3a protein (Figure 5D), and the reporter gene with PPP2R1B showed significant lower activities in the eIF3a knockdown cells compared with control cells (Figure 5E). Furthermore, HCQ, an autophagy inhibitor, or MG132, a proteasome inhibitor, had no effect on the downregulation of PPP2R1B in the cells with eIF3a knockdown (Figures 5F,G). Based on these results, we deduced that eIF3a regulated the translation of PPP2R1B.

### The Association of eIF3a and PPP2R1B is Validated in Samples From Patients With Melanoma

**FIGURE 6:**
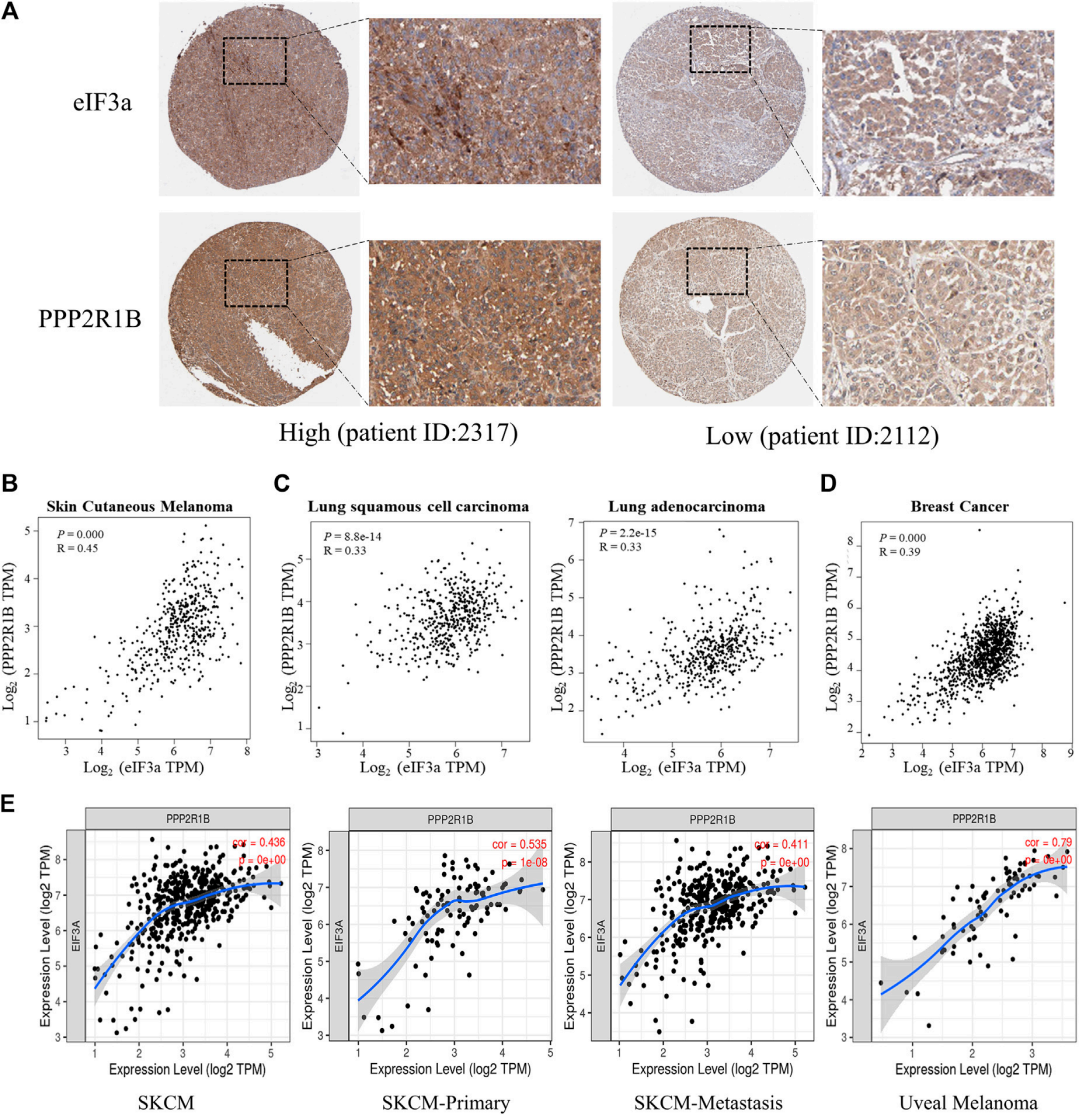
The association of eIF3a and PPP2R1B is validated in samples from patients with melanoma. **(A)** IHC analyses of eIF3a and PPP2R1B levels in melanoma tissues from the HPA database. **(B)** Pearson’s correlation analyses of eIF3a and PPP2R1B mRNA levels in melanoma (data from TCGA). **(C)** Pearson’s correlation analyses of eIF3a and PPP2R1B mRNA levels in lung cancer. **(D)** Pearson’s correlation analyses of eIF3a and PPP2R1B mRNA levels in breast cancer. **(E)** Pearson’s correlation analyses of the eIF3a and PPP2R1B mRNA levels in primary, metastatic, and uveal melanoma. TPM represents transcripts per million.

To further validate the association between eIF3a and PPP2R1B in human melanoma patient samples, we first assessed protein expression levels of eIF3a and PPP2R1B by the Human Protein Atlas (https://www.proteinatlas.org/). The results showed that the expression of eIF3a was positively correlated with PPP2R1B expression in patients with melanoma (Figure 6A and Supplementary Figure S4). We further analyzed the correlation between eIF3a and PPP2R1B expression in TCGA database. Consistently, the strong positive correlation between eIF3a and PPP2R1B expression was observed in melanoma, lung cancer and breast cancer (Figures 6B–D). In addition, we also analyzed the relationship between eIF3a and PPP2R1B expression in primary, metastatic and uveal melanoma using the TIMER web server (https://cistrome.shinyapps.io/timer/), and found that eIF3a expression was positively correlated with PPP2R1B expression (Figure 6E).

## Discussion

**FIGURE 7:**
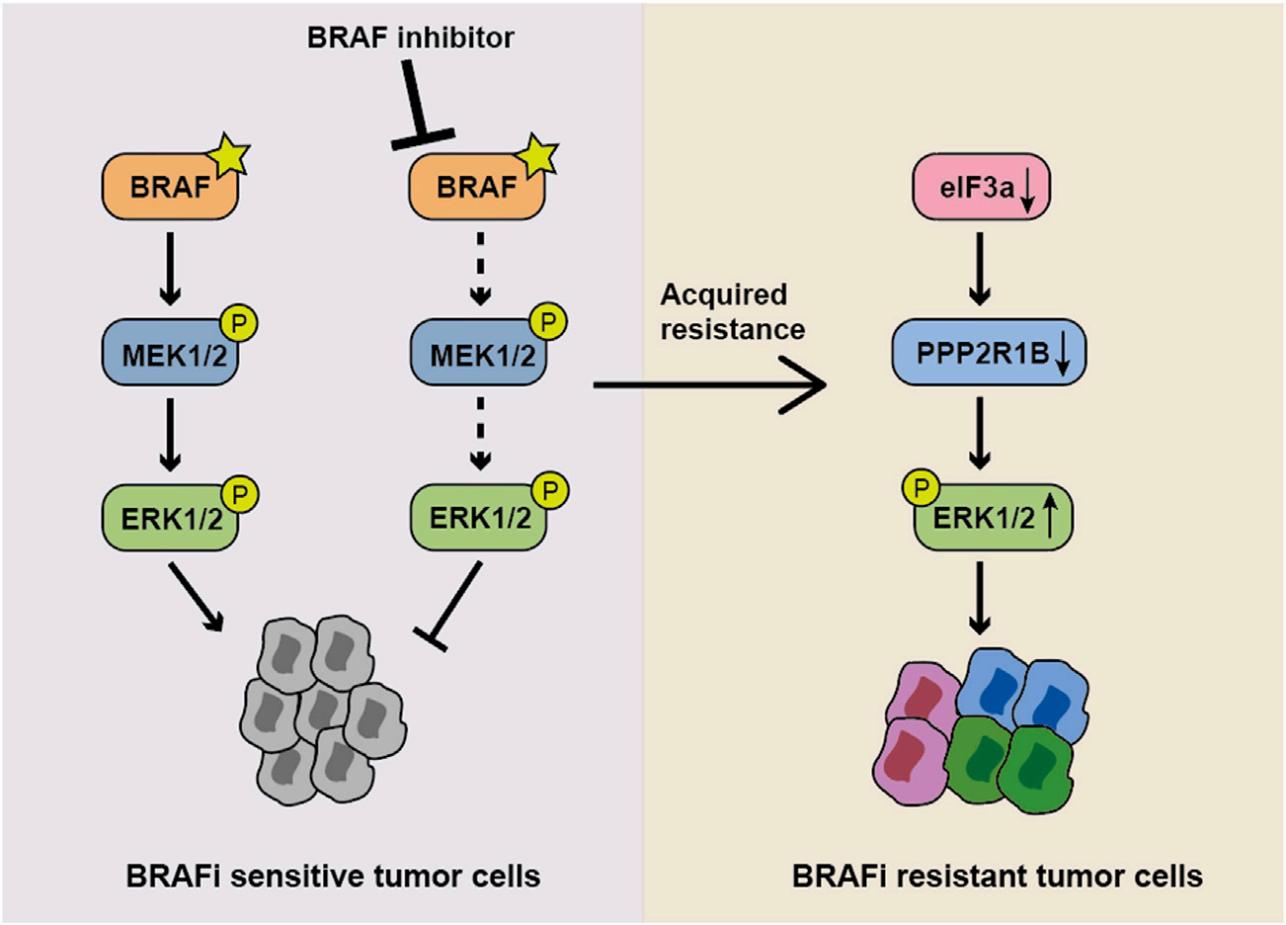
Model showing the role of the eIF3a-PPP2R1B-ERK axis in regulating BRAF inhibitor resistance. In normal BRAFV600E mutant cells, the BRAF inhibitor suppresses ERK activity and cell proliferation. When eIF3a is depleted, PPP2R1B translation is inhibited, resulting in the persistence of p-ERK and the subsequent occurrence of drug resistance.

Acquired resistance to vemurafenib is a major hurdle in the management of melanoma; therefore, studies exploring the mechanism underlying vemurafenib resistance and developing more effective therapeutic strategies for patients with melanoma carrying the BRAFV600E are imperative. In this study, we revealed for the first time the potentially important role of eIF3a in targeted therapy. We found that eIF3a was downregulated in vemurafenib-resistant A375 cells, and its depletion significantly decreased the response of melanoma cells to BRAF inhibitors. Furthermore, eIF3a modulated the sensitivity of melanoma cells to vemurafenib by promoting the translation of PPP2R1B, and PPP2R1B further resulted in decreased ERK activity by dephosphorylating this kinase, indicating that the eIF3a/PPP2R1B/ERK axis was a key mediator of melanoma resistance to vemurafenib (Figure 7). The positive relationship between eIF3a and PPP2R1B expression was also observed in melanoma tissues. These results suggested that eIF3a may be a predictor to evaluate the efficacy of BRAF inhibitors.

As a key regulator in cancer, eIF3a is expressed at high levels in multiple cancers and is relevant to the sensitivity of DNA damage-induced therapy, such as platinum agents (Zhang Y. et al., 2015) and ionizing radiation (Tumia et al., 2020). As shown in our previous study, eIF3a increases the sensitivity of ovarian cancer and non-small cell lung cancer to platinum-based chemotherapy, and the patients with a high level of eIF3a have a better prognosis (Yin et al., 2011b; Zhang Y. et al., 2015).

ERK activation is critical for cell proliferation and differentiation, and even alters the response of cancer cells to chemotherapies and targeted therapies (Marshall, 1995). In addition, ERK regulates the metabolic processes of cancer cell, such as glucose metabolism and fatty acid metabolism, and promotes cancer cell invasion and migration (Lee et al., 2020). As a critical mechanism significantly affecting cancer pathogenesis, the regulation of ERK has been extensively investigated. Levels of activated ERK are regulated by multiple proteins or miRNAs, including BOP1, VRK3, and miR-30 (Han and Kim, 2020; Wang et al., 2020). Therefore, a better understanding of ERK-mediated drug resistance may be helpful for in-depth studies of the antitumor mechanism. Here, we showed that reduced eIF3a expression caused ERK activation, as evidence by increased ERK phosphorylation, thus leading to vemurafenib resistance, which may provide novel strategy to rescue drug sensitivity for anticancer drug development.

We performed a proteomics analysis using mass spectrometry to identify eIF3a interacting proteins and illustrate the regulatory mechanism of eIF3a in ERK activation. The expression of PPP2R1B, a phosphatase, was decreased in cells subjected to eIF3a knockdown. Notably, eIF3a knockdown or overexpression decreased or increased the expression of the PPP2R1B protein, respectively. In addition, consistent with other phosphatases of ERK, PPP2R1B knockdown in melanoma cell lines led to increased ERK phosphorylation and mediated the negative regulatory effect of eIF3a on vemurafenib sensitivity. As a tumor suppressor, mutations and alterations in PPP2R1B have been found in human cancers, including colon cancer, lung cancer and cervical cancer, and is involved in chemotherapy sensitivity (Wang et al., 1998; Tamaki et al., 2004; Yeh et al., 2007). Previous studies have reported that silencing PPP2R1B enhances 5-FU resistance (Zhang et al., 2015), and PPP2R1B overexpression increases the sensitivity of tongue squamous cell carcinoma and esophageal cancer cells to docetaxel and cisplatin, respectively by acting as an AKT phosphatase (Hamano et al., 2011; Cui et al., 2020). In the present study, PPP2R1B influenced vemurafenib sensitivity by inhibiting ERK phosphorylation, and ectopic expression of PPP2R1B restored cell sensitivity to vemurafenib. Thus, ERK may be a novel protein target of PPP2R1B phosphatase, and PPP2R1B may be a potential target for developing novel strategies against vemurafenib resistance.

Last, we also observed strong correlations between eIF3a and PPP2R1B expression between tumor tissues from HPA and TCGA databases, further supporting our findings in cells. In addition, the correlation between eIF3a and PPP2R1B was also observed in lung cancer and breast cancer tissue, suggesting that the mechanism is universal. Due to the importance of vemurafenib in clinical applications, studies investigating the regulatory axis of eIF3a-PPP2R1B-ERK are crucial and worthwhile. Moreover, our findings highlight eIF3a as a promising biomarker for melanoma or other cancers to predict the therapeutic effect of vemurafenib.

Taken together, our results revealed that the eIF3a-PPP2R1B-ERK axis may contribute to vemurafenib resistance, which provided new insights into not only vemurafenib resistance but also the prediction of the response to vemurafenib treatment.

## Data Availability

The datasets presented in this study can be found in online repositories. The names of the repository/repositories and accession number(s) can be found in the article/Supplementary Material.

## Abbreviations

eIF3a, eukaryotic translation initiation factor 3a; ERK, p44/42 MAPK (Erk1/2); PPP2R1B, protein phosphatase 2 scaffold subunit Abeta; RIP, RNA immunoprecipitation; TCGA, the cancer genome atlas; GEO, gene expression omnibus; qPCR, quantitative real-time PCR.
