# Supplementary figures and images for: The Downregulation of eIF3a Contributes to Vemurafenib Resistance in Melanoma by Activating ERK via PPP2R1B
Source: Front Pharmacol. 2021 Aug 27;12:720619. doi: 10.3389/fphar.2021.720619 (PMC8430041; doi:10.3389/fphar.2021.720619)

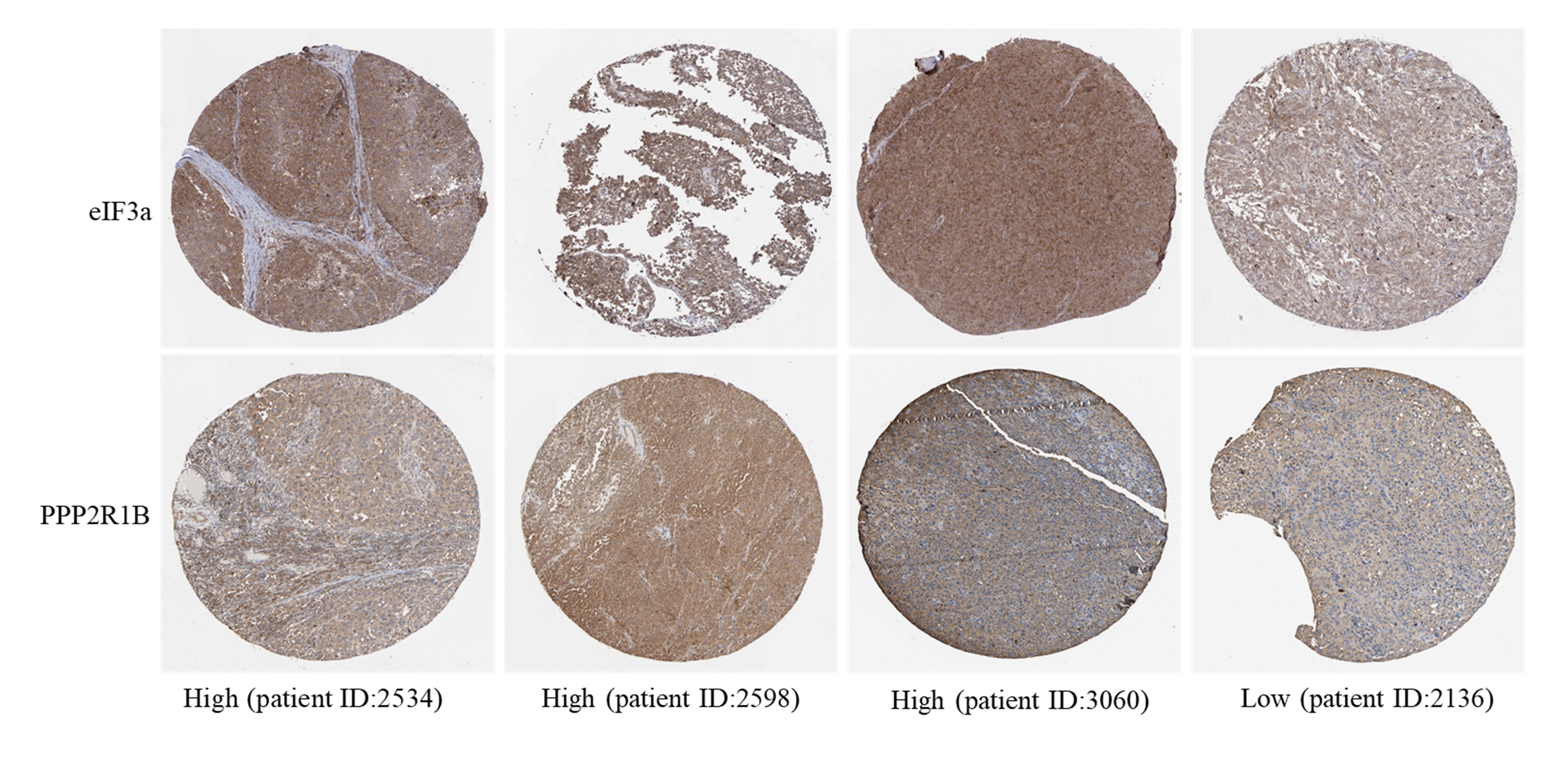

Supplement: Supplementary file 3 [file Image4.JPEG]

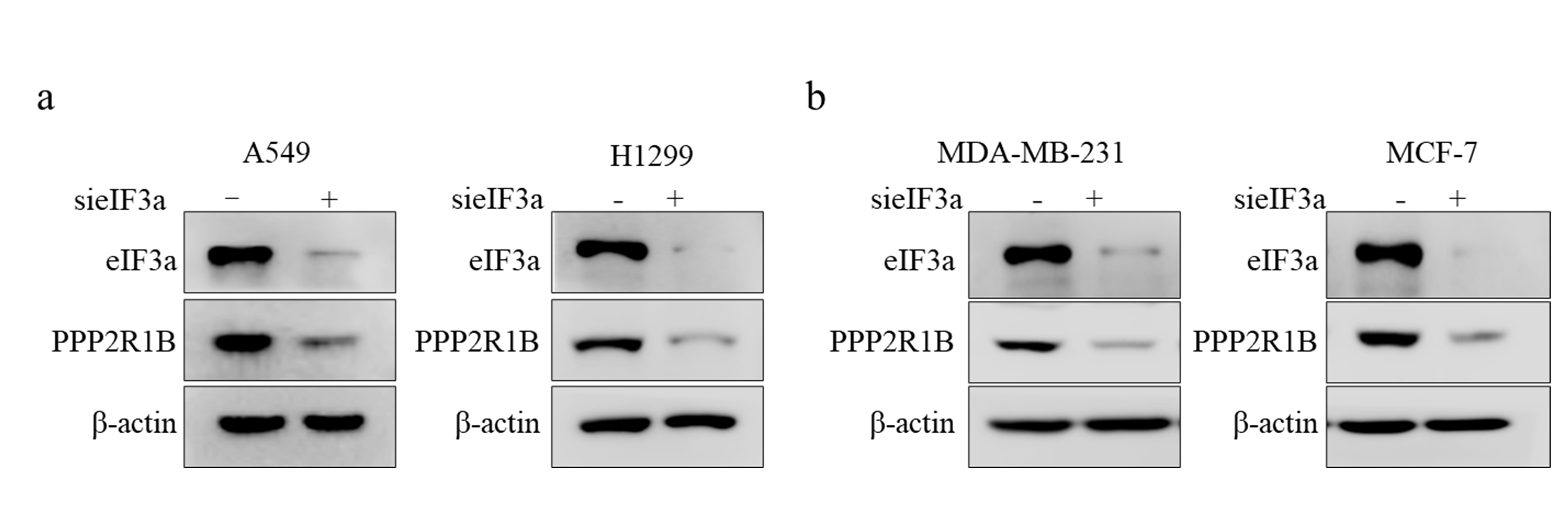

Supplement: Supplementary file 4 [file Image2.JPEG]
